# Pharmacokinetic and pharmacodynamic modelling after subcutaneous, intravenous and buccal administration of a high-concentration formulation of buprenorphine in conscious cats

**DOI:** 10.1371/journal.pone.0176443

**Published:** 2017-04-26

**Authors:** Graeme M. Doodnaught, Beatriz P. Monteiro, Javier Benito, Daniel Edge, Francis Beaudry, Ludovic Pelligand, Paulo Steagall

**Affiliations:** 1 Département de Sciences Cliniques, Faculté de Médecine Vétérinaire, Université de Montréal, Saint-Hyacinthe, Québec, Canada; 2 Groupe de Recherche en Pharmacologie Animal du Québec (GREPAQ), Département de Biomédecine Vétérinaire, Faculté de Médecine Vétérinaire, Université de Montréal, St-Hyacinthe, Québec, Canada; 3 Zoetis Inc., Florham Park, New Jersey, United States of America; 4 Department of Clinical Sciences and Services, The Royal Veterinary College, North Mymms, Hertfordshire, United Kingdom; University of Bari, ITALY

## Abstract

**Background:**

The aim of this study was to describe the joint pharmacokinetic-pharmacodynamic model and evaluate thermal antinociception of a high-concentration formulation of buprenorphine (Simbadol^™^) in cats.

**Methods:**

Six healthy cats (4.9 ± 0.7 kg) were included in a prospective, randomized, blinded, crossover study. Simbadol^™^ (1.8 mg mL^-1^) was administered by the subcutaneous (SC; 0.24 mg kg^-1^), intravenous (IV; 0.12 mg kg^-1^) or buccal (OTM; 0.12 mg kg^-1^) route of administration and thermal thresholds (TT) were compared with a saline group (SAL). Thermal threshold testing and blood sampling were performed at predetermined time points up to 72 hours including a placebo group. Plasma buprenorphine and norbuprenorphine concentrations were measured using liquid chromatography mass spectrometry. A bespoke bicompartmental pharmacokinetic model simultaneously fitted data from two analytes/three routes of administration. Temporal changes in TT were analyzed using one-way ANOVA followed by Dunnett’s test and treatment comparisons using two-way ANOVA with Bonferroni’s correction (P < 0.05).

**Results:**

Thermal thresholds were significantly increased after SC, IV and OTM from 1–24 hours (except 2 hours), 0.5–8 hours (except 6 hours), and 1–8 hours (except 6 hours), respectively, when compared with baseline. Thermal thresholds were significantly increased after SC (1–30 hours), IV (1–8 hours) and OTM (1–12 hours) when compared with SAL, but not different among buprenorphine-treated cats. The absolute buprenorphine clearance was 0.98 L kg^-1^ hour^-1^, volume of distribution at steady state was 7.9 L kg^-1^ and the elimination-half-life was 12.3 hours. Bioavailability for SC and OTM was 94% and 24%, respectively. Subcutaneous absorption was biphasic. An initial peak (0.08 hours) was followed by a slow (half-life 11.2 hours) and progressive (peak acceleration at 2.8 hours) uptake.

**Conclusion:**

The SC administration of Simbadol^™^ was characterized by prolonged absorption half-life and sustained plasma concentrations yielding long-lasting antinociception (≥ 24 hours) when compared with the IV and OTM routes.

## Introduction

Buprenorphine is an opioid analgesic drug that is commonly administered for the treatment of feline perioperative pain. The use of buprenorphine in this species has been recently reviewed in both experimental and clinical setting [1]. At standard clinical doses (0.02 mg kg^-1^) and concentrations (0.3 mg mL^-1^), buprenorphine is poorly absorbed and has limited antinociceptive effect after subcutaneous (SC) administration [2]. Further investigation showed that increased doses (> 1.2 mg kg^-1^) administered by this route of administration can provide prolonged antinociception and improved absorption compared with standard doses [3]. Simbadol^™^ (1.8 mg mL^-1^, buprenorphine hydrochloride; Zoetis, NJ, USA) is an FDA-approved high-concentration formulation of buprenorphine for cats. The drug is indicated for the control of postoperative pain and approved for subcutaneous only administration at 0.24 mg kg^-1^ every 24 hours up to three days. There is an interest in investigating the antinociceptive effects, pharmacokinetics (PK) and pharmacodynamics (PD) of this high-concentration of buprenorphine after SC, buccal or intravenous (IV) administration in conscious cats. Using a joint PK model of the three routes of administration would provide more robust estimates of the PK parameters of Simbadol^™^ when compared with traditional PK methods analyzing each route of administration separately [4].

The aims of this study were to 1) describe a joint PK modelling of Simbadol^™^ in awake cats, 2) evaluate the time-course of thermal antinociception in the same individuals, and 3) estimate PD parameters by PK-PD modeling. It was hypothesized that 1) joint PK would provide a robust method for understanding PK modeling, 2) Simbadol^™^ would produce dose-dependent thermal antinociception and 3) PK-PD modeling would explain how plasma concentrations of buprenorphine and thermal antinociception are correlated after administration of Simbadol^™^. This study reports joint PK modelling and PK-PD modelling of buprenorphine after the administration of Simbadol^™^.

## Materials and methods

### Animals

The animal care committee of the Université de Montréal approved the study protocol (14-Rech-1761). This study is reported according to the ARRIVE guidelines [5].

Six healthy adult domestic short haired cats (4.9 ± 0.7 kg, four males and two females) were included in the study. Cats were purchased from another research laboratory which they had been used as controls for another study. They were all adopted at the end of the study. The cats were group housed in a long-term accommodation (room) with temperature (20–22°C) and humidity (40–70%) control, and according to the Canadian Council for Animal Care guidelines.

The cats were fed a commercially available diet twice daily with *ad libitum* water. All cats were healthy based on physical examination, hematology and serum chemistry profile. Environmental enrichment was provided following the American Association of Feline Practitioners and International Society of Feline Medicine guidelines [6]. During testing, cats were housed individually in stainless steel adjacent cages (67 x 55 x 68 cm^3^). Body weight was monitored on a weekly basis. Cats were acclimated to the testing procedures several weeks before the study began (S1 File—Appendix 1).

### Experimental design

The experimental phase was divided into two parts.

Phase I (Thermal thresholds after saline 0.9%)—Cats were administered sterile saline 0.9% (NaCl 0.9%; Baxter, ON, Canada) by the subcutaneous route of administration (same volume as 0.24 mg kg^-1^ dose of Simbadol^™^) between the shoulder blades, and thermal thresholds (TT) were evaluated for up to 72 hours (see time points below). Blood collection or venous catheterization was not performed on Phase I to avoid unnecessary stress to the cats.

Phase II (Cross-over thermal threshold testing and blood sampling)—Phase II used a randomized, prospective, blinded, crossover study design with a 14-day minimum wash-out period between treatments. Approximately 12 hours before TT testing, a short-term catheter was introduced in the cephalic vein and general anesthesia was induced and maintained using 8 mg/kg of propofol (Diprivan; AstraZeneca, ON, Canada) and isoflurane (Isoflurane, Aerrane; Baxter, ON, Canada) in 100% oxygen, respectively. A central venous catheter (Peel Away Single Lumen 19Ga, PI-1910; Mila International, KY, USA) was aseptically introduced in the jugular vein, sutured, and covered with light soft bandage. The jugular catheter was used for *blood sampling* throughout the study and removed after the testing period. The cephalic catheter was maintained in all cats until *treatment administration* and was used for administration of the test drug by the IV route when applicable. It was removed immediately after treatment administration. At the end of the procedure, cats were allowed to recover from anesthesia in a calm and quiet environment.

Cats were randomly assigned to receive one of the following treatments: a. Simbadol^™^ by the intravenous route (IV, 0.12 mg kg^-1^) via cephalic catheter followed by flush with saline; b. Simbadol^™^ by the subcutaneous route (SC, 0.24 mg kg^-1^). The drug was injected between the shoulder blades; and c. Simbadol^™^ by the buccal route (OTM, 0.12 mg kg^-1^). The volume of buprenorphine was slowly administered into the cheek pouch using a 1mL syringe in the contralateral side to the central venous catheter position. Doses for the IV and OTM routes were based on previous safety and efficacy studies performed by the manufacturer, and clinical interest. Randomization was performed using an online software (www.randomization.com). Randomization and treatment administration were performed by two individuals who were not involved with TT testing (PS/GD).

### Measurements of thermal threshold

Antinociception was evaluated using a wireless TT device (WTT1, Topcat Metrology Ltd, UK). The test has been validated in free-ranging cats [7] and several studies using the TT device and evaluating the antinociceptive effects of buprenorphine have been reported in the species [3, 8–16]. The equipment was calibrated and maintained according to the manufacturer’s recommendations. The TT device is incorporated into an elasticated vest, containing a wireless receiver, power supply, LCD display and thermal probe which is applied around the thorax of a free roaming cat. An adjustable air bladder is used to maintain consistent pressure and direct contact of the temperature probe with the shaved lateral thorax. For each measurement, the skin temperature (ST) is recorded prior to thermal stimulus. For TT testing, the evaluator triggers a ramped heat stimulus (0.6°C second^−1^) using a hand-held device which is stopped once the cat exhibits a behavioral response (e.g. vocalization, rolling, jumping, etc.; considered the TT), or when the cut-off of 55°C is reached. If cut-off was reached, this value (55°C) was used as the TT. A single evaluator (BM) performed TT testing and was blinded to the treatments. Thermal threshold testing was evaluated before (baseline; time 0) and at 0.5, 1, 2, 4, 6, 8, 12, 24, 30, 36, 48, 60 and 72 hours after treatment administration for both phases. At each testing day and 30 minutes after placement of TT device, baseline values were determined using the mean of three recordings performed at 15-minute intervals prior to treatment.

Any behavioral changes, adverse reactions or additional observations were recorded during the testing period. Oral pH was measured at baseline (pre-treatment) and at 48 hours after treatment.

### Blood sampling

During Phase II, blood sampling was performed from the jugular vein via central venous catheter after 2 and 8 min of treatment administration, and after each TT testing. The volume of blood collected was adjusted for each individual so that less than 10% of the cat’s total blood volume was removed over the study period (2 mL maximum per sample). After each collection, the catheter was flushed with 1 mL of heparinized saline and the injection plug was changed to minimize contamination of subsequent sampling. Blood was transferred to K_3_EDTA tubes and centrifuged at 2000 *g* for 10 minutes. Plasma was separated and stored at -80°C before buprenorphine/norbuprenorphine HPLC-MS/MS analysis.

### Buprenorphine and norbuprenorphine assay

Appendix 2 (S2 File) provides description of the analytical method for buprenorphine and norbuprenorphine using high performance liquid chromatography-mass spectrometry (HPLC-MS/MS). The method met standards for sensitivity, linearity, precision, accuracy and stability generally accepted in bioanalytical chemistry [17]. Limits of quantification were 0.1 to 100 ng mL^-1^ for buprenorphine and 0.2 to 100 ng mL^-1^ for norbuprenorphine.

### Joint PK and PK-PD modeling

Population pharmacokinetic modelling was performed with Phoenix NMLE^®^, version 1.3, Certara (Princeton, NJ, USA) installed on a Dell Precision 7510 computer (core i7). Full description of the joint population PK and PK-PD model is provided in Appendix 3 (S3 File). Briefly, a two compartmental model was used to simultaneously model the plasma concentration-time curves of buprenorphine and norbuprenorphine (formed through conversion from buprenorphine and first pass effect). The SC, IV and OTM administration routes were included jointly in the PK model to increase the number of degree of freedom. In order to explore the biphasic nature of the SC absorption, several complex candidate models were elaborated and compared. Estimates of PK variables were provided with inter-individual variability (IIV%). Population PD parameters were estimated by sequential population PK-PD modeling. The best PD model was selected among a series of candidate models including negative hysteresis and antinociceptive effects of buprenorphine with or without norbuprenorphine.

### Statistical analysis

Prospective power analysis concluded that a sample size of six cats would be sufficient to detect mean temperature differences of > 3.2°C with a power of 0.8 and an alpha level set at 0.05 based on a previous study [18]. Statistical analyses were performed using a software (GraphPad Prism, GraphPad software Inc., California, USA). Thermal threshold values were used as the outcome variable for comparisons. Thermal thresholds for each treatment were analyzed for temporal changes using one-way ANOVA for repeated measures followed by the Dunnett’s test when appropriate. Treatment comparisons were made using two-way ANOVA followed by Bonferroni’s correction (*p* < 0.05). Data were expressed as mean ± SD.

## Results

Adverse effects and behavioral changes—Adverse effects were not observed in Phase I. Overall, signs of euphoria (rolling, kneading with thoracic paws, meowing, and purring) and agitation were recorded after treatment with all routes/cats in Phase II. Vomiting and diarrhea were observed in two cats during the study; one in the IV group (diarrhea on day 2 and vomiting once on day 3) and one in the OTM group (diarrhea only on day 3). Both cats remained bright and alert and their clinical signs spontaneously resolved. Short-term hypersalivation was noted in two cats immediately following OTM treatment. Dysphoria was not observed in this study. Mean ± SD of oral pH before and after (48 hours) all treatments were 8.8 ± 0.4 and 8.7 ± 0.5, respectively.

Skin temperature and thermal thresholds—Mean ± SD baseline ST for all treatments (Phase I and II) was 36.9 ± 0.6°C. Skin temperature (ST) was not significantly increased after IV, OTM or saline 0.9% treatments when compared with baseline. Skin temperature was significantly increased in SC treatment between 60 and 720 minutes (except 120 and 360 minutes; *p* = 0.0001). These values were within normal range and hyperthermia (ST > 39.5°C) was not observed. Skin temperature was significantly increased from 0 to 3600 minutes (except 1440 to 2160 minutes) after IV treatment, from 0 to 4320 minutes (except 30 and 1800 to 3600 minutes) after SC treatment, and from 0 to 4320 minutes (except 720 to 2160 minutes) after OTM treatment (*p* < 0.05) when compared with saline 0.9% (Table 1).

**Table 1:** Skin temperature (mean ± SD) in cats after saline (SAL) or buprenorphine administration via the three treatment routes: Intravenous (IV), subcutaneous (SC), and buccal (OTM).

	Route	Time (hours)													
		0	0.5	1	2	4	6	8	12	24	30	36	48	60	72
Variable ST (°C)	SAL (n = 6)	36.3 ± 0.4	36.4 ± 0.3	36.5 ± 0.1	36.2 ± 0.2	36.2 ± 0.2	36.3 ± 0.2	36.3 ± 0.3	36.4 ± 0.3	36.5 ± 0.2	36.5 ± 0.5	36.6 ± 0.4	36.4 ± 0.4	36.4 ± 0.3	36.4 ± 0.3
	IV (n = 6)	37.3 ± 0.5*	37.4 ± 0.8*	37.8 ± 0.5*	37.5 ± 0.7*	37.7 ± 0.5*	37.4 ± 0.3*	37.2 ± 0.2*	37.5 ± 0.6*	37.2 ± 0.4	36.9 ± 0.3	37.2 ± 0.3	37.1 ± 0.4*	37.1 ± 0.3*	36.8 ± 0.4
	SC (n = 6)	37.0 ± 0.3*	37.1 ± 0.6	37.7 ± 0.4†*	37.5 ± 0.5*	37.6 ± 0.8†*	37.6 ± 0.5*	37.6 ± 0.8†*	37.6 ± 0.4†*	37.3 ± 0.5*	37.1 ± 0.5	37.2 ± 0.6	37.0 ± 0.3	37.1 ± 0.2	37.1 ± 0.4*
	OTM (n = 6)	37.2 ± 0.4*	37.4 ± 0.5*	37.7 ± 0.3*	37.4 ± 0.3*	37.5 ± 0.4*	37.4 ± 0.4*	37.1 ± 0.3*	37.0 ± 0.5	37.2 ± 0.2	37.1 ± 0.3	37.1 ± 0.4	37.2 ± 0.3*	37.3 ± 0.5*	37.3 ± 0.4*

ST (Skin Temperature); SAL (Saline SC); IV (0.12 mg kg^-1^ buprenorphine IV); SC (0.24 mg kg^-1^ buprenorphine SC); OTM (0.12 mg kg^-1^ buprenorphine buccal route of administration).

^†^ Significant difference from baseline values.

* Significant difference from saline treatment (Two-way ANOVA).

Mean ± SD baseline TT for all treatments (Phase I and II) was 45.3 ± 1.7°C. Thermal thresholds were not significantly different among IV, SC and OTM treatments. Thermal thresholds were significantly increased after IV treatment between 30 and 480 minutes (except 360 minutes; *p* < 0.001), after SC treatment between 60 and 1440 minutes (except 120 minutes; *p* < 0.001), and after OTM treatment between 60 and 480 minutes (except 360 minutes; *p* < 0.001) when compared with baseline values. Thermal thresholds were significantly increased after IV treatment from 30 to 480 minutes, after SC treatment from 60 to 1800 minutes, and after OTM from 60 to 720 minutes when compared with saline 0.9% (*p* < 0.05). Thermal thresholds did not increase after treatment with saline 0.9% (Table 2).

**Table 2:** Thermal threshold (mean ± SD) in cats after saline (SAL), or buprenorphine administration via the three treatment routes: Intravenous (IV), subcutaneous (SC), and buccal (OTM).

	Route	Time (hours)													
		0	0.5	1	2	4	6	8	12	24	30	36	48	60	72
Variable TT (°C)	SAL (n = 6)	43.8 ± 2.1	44.7 ± 2.7	44.2 ± 2.8	43.8 ± 1.3	43.5 ± 2.0	43.5 ± 2.0	43.8 ± 2.4	44.0 ± 2.2	44.2 ± 2.2	44.0 ± 2.5	45.0 ± 3.3	44.6 ± 1.8	43.8 ± 1.7	44.0 ± 1.8
	IV (n = 6)	45.7 ± 1.8	50.3 ± 3.7†*	49.6 ± 2.4†*	50.2 ± 3.3†*	49.9 ± 2.0†*	48.9 ± 5.0*	50.2 ± 5.0†*	46.4 ± 2.2	48.5 ± 4.4	46.2 ± 2.2	46.7 ± 2.5	45.1 ± 1.9	45.7 ± 2.4	46.7 ± 2.6
	SC (n = 6)	45.6 ± 1.1	49.4 ± 2.3	49.7 ± 4.1†*	49.2 ± 3.1*	50.2 ± 4.6†*	50.9 ± 3.7†*	51.1 ± 4.2†*	49.7 ± 3.1†*	49.7 ± 3.3†*	49.3 ± 3.2*	49.0 ± 2.8	48.0 ± 4.0	45.1 ± 1.7	45.8 ± 1.6
	OTM (n = 6)	46.1 ± 1.6	49.3 ± 4.8	49.5 ± 4.8†*	51.2 ± 3.9†*	50.5 ± 3.7†*	48.6 ± 4.2*	50.4 ± 4.3†*	49.4 ± 3.8*	47.7 ± 2.2	46.8 ± 1.3	47.1 ± 1.2	46.0 ± 1.2	46.7 ± 1.4	46.4 ± 1.2

TT (Thermal threshold); SAL (Saline SC); IV (0.12 mg kg^-1^ buprenorphine IV); SC (0.24 mg kg^-1^ buprenorphine SC); OTM (0.12 mg kg^-1^ buprenorphine buccal route of administration).

^†^ Significant difference from baseline values.

* Significant difference from saline (Two-way ANOVA).

**Fig 1:**
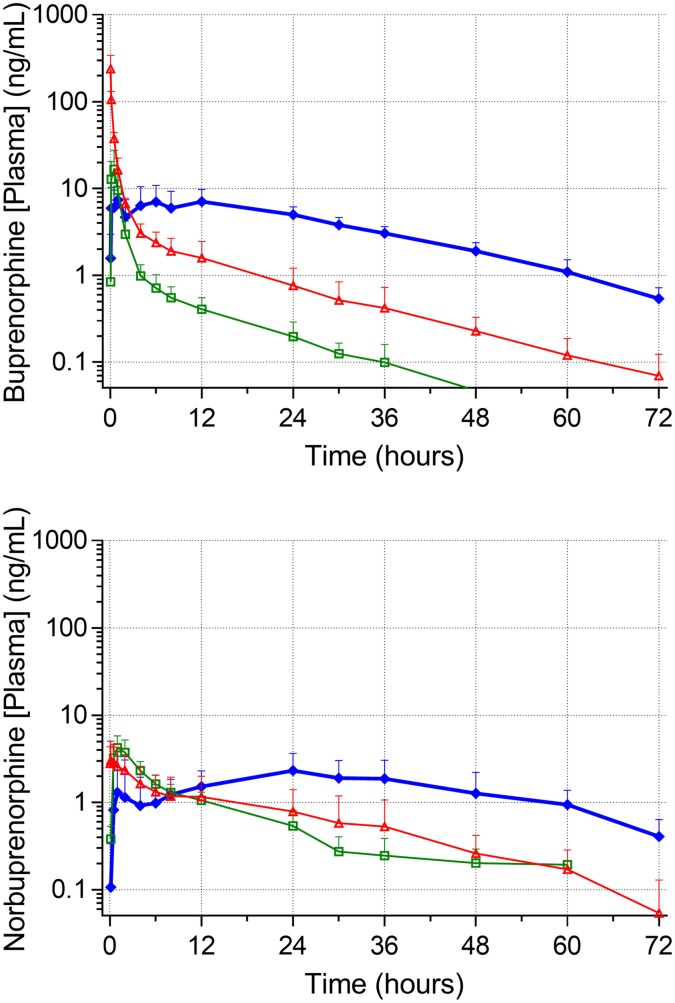
Mean plasma concentrations of buprenorphine and norbuprenorphine (± SD) in six conscious cats. IV (0.12 mg/kg buprenorphine IV, red triangles); SC (0.24 mg/kg buprenorphine SC, blue diamonds); OTM (0.12 mg/kg buprenorphine buccal route of administration, green squares); [Plasma] (Plasma concentration).

Blood sampling—A central jugular catheter was removed by one cat at 24 hours after IV administration of Simbadol^™^. Fig 1 shows mean plasma concentration profiles for buprenorphine and norbuprenorphine (Fig 1)

**Fig 2:**
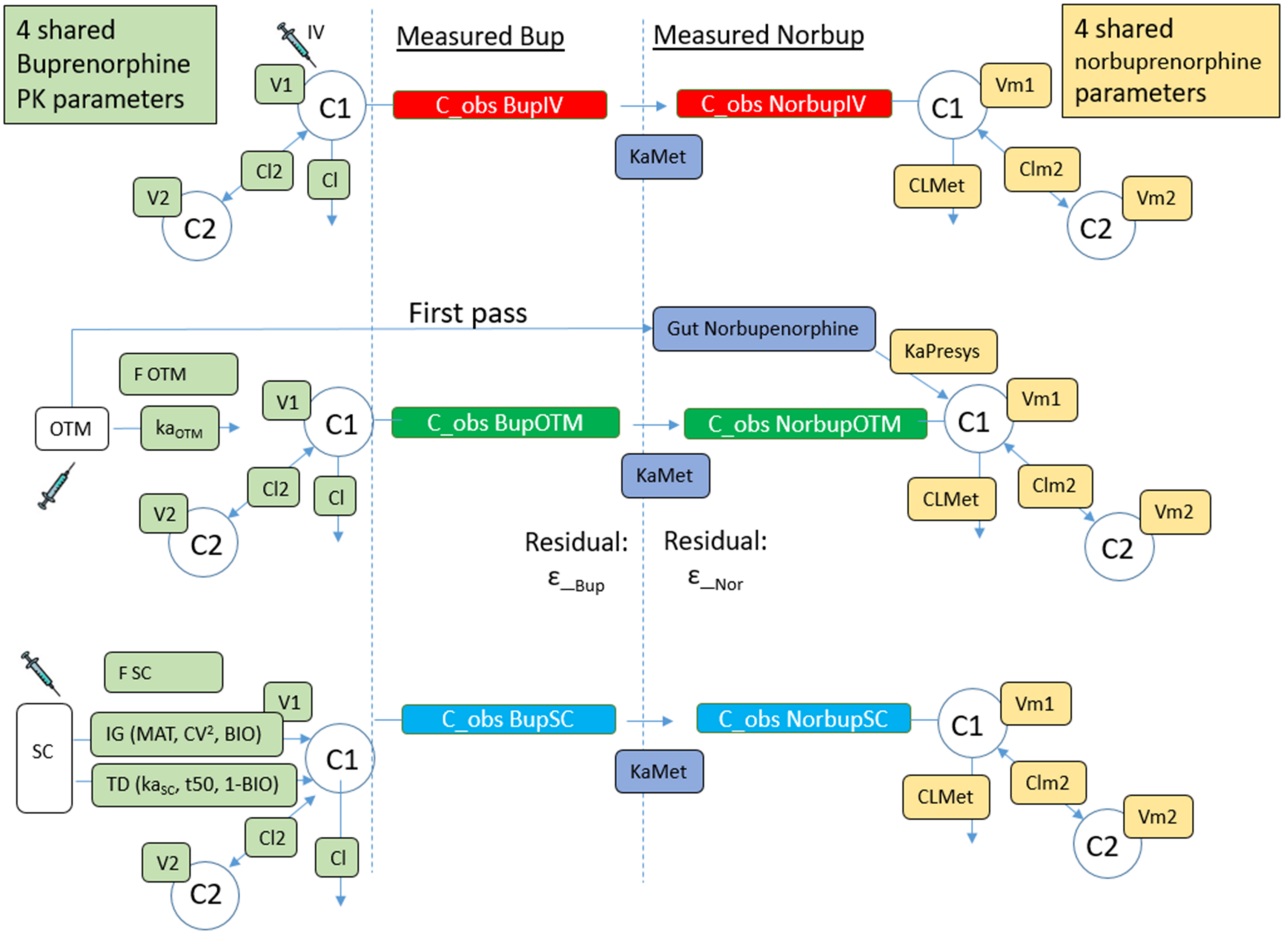
Pharmacokinetic-pharmacodynamic (PK-PD) model representation for buprenorphine and norbuprenorphine after subcutaneous, intravenous and buccal administration in six cats. For the SC route, combined Inverse Gaussian (IG, rapid but short lasting) and Time-dependent (TD, delayed and progressive onset) inputs. Buprenorphine central PK parameters; clearance (CL), volume of distribution of the central compartment (V1), intercompartmental clearance (CL2), volume of distribution of peripheral compartment (V2). Norbuprenorphine central PK parameters: clearance (CLMet), volume of distribution of the central compartment (V1Met), intercompartmental clearance (CL2Met) and volume of distribution of the peripheral compartment (V2 Met). Rate constant of transformation from parent to metabolite (KaMet), first pass norbuprenorphine absorption rate (Kafirst-pass). PK parameters specific to OTM route: bioavailability (FOTM, parent and metabolite), absorption rate constant (kaOTM). PK parameters specific to SC route: bioavailability (FSC), proportion taken by IG input (BIO) and time-dependent delayed input (1-BIO), mean input rate time (MAT) was 7.21 h (3.5%) and variance of the input time (CV), maximal absorption rate constant (kaSC), time to achieve 50% of this maximum rate (T50).

Pharmacokinetic modeling and parameters—Fig 2 shows the structure of the final population PK model for buprenorphine and norbuprenorphine (Fig 2). For all parameters listed below, the inter-individual variability (IIV %) is reported immediately following each estimate where appropriate.

For buprenorphine, a model that best predicted the individual observed plasma concentrations after SC route (see S3 File—Appendix 3), and that has been previously used for systemic absorption of local anesthetic after perineural administration (16), was used. This model combined Inverse Gaussian (IG, rapid but short duration) and Time-dependent (TD, delayed and progressive onset) inputs. The three routes of administration shared four central PK parameters; clearance (CL = 0.98 L kg^-1^ hour^-1^, 2.4%), volume of distribution of the central compartment (V1 = 0.75 L kg^-1^, 11.3%), intercompartmental clearance (CL2 = 0.70 L kg^-1^ hour^-1^, 0%) and peripheral volume of distribution (V2 = 7.15 L kg^-1^, 8.1%) with a common proportional residual error term. The total body clearance of buprenorphine was moderate to high according to Toutain et al. 2004 [19]. Volume of distribution at steady-state (Vdss) was 7.89 L kg^-1^. The average beta elimination half-life for this bicompartmental model was 12.3 hours [20].

For PK parameters, specific to the OTM treatment, the bioavailability F_OTM_ was 23.6% (IIV 25%) and the absorption rate constant (ka_OTM_) was 1.67 hour^-1^ (25.1%), yielding an absorption half-life of 0.42 hours. For the SC treatment, the total bioavailability F_SC_ was 94% (IIV 23.4%). A proportion of 10.1% (IIV 113%) of F_SC_ was absorbed through early uptake (BIO = proportion taken by IG input) and the rest (1-BIO = 89.1%) was absorbed through a time-dependent delayed input (TD). For the IG input of the SC absorption, mean input rate time (MAT) was 7.21 hours (3.5%) and variance of the input time (CV) was 5.46 (0.5%). This translated into an initial peak of plasma concentration observed at 0.08 hours (5 minutes, at T_max_, the mode of the IG function) corresponding to 10% of the buprenorphine being rapidly absorbed. For the TD input, the maximal absorption rate constant (ka_SC_) was 0.062 hour^-1^ (14.9%), yielding a slow late absorption half-life (11.2 hours). The time to achieve 50% of this maximum rate (T_50_) was 2.8 hour (27%).

For norbuprenorphine, it was thought that it was exclusively generated from plasma buprenorphine degradation, with Ka_Met_ the irreversible conversion rate constant from the parent drug to its metabolite. The PK of norbuprenorphine was best described using a two compartment model for which four parameters common to the three administration routes could be estimated: norbuprenorphine clearance (CL_Met_ = 0.42 L kg^-1^ hour^-1^, IIV 4.3%), volume of distribution of the central compartment (V1_Met_ = 0.323 L kg^-1^, 0.1%), intercompartmental clearance (CL2 _Met_ = 7.662 L kg^-1^ hour^-1^) and volume of distribution of the peripheral compartment (and V2 _Met_ = 4.69 L kg^-1^). Random effects for CL2 _Met_ and V2 _Met_ were not estimated. In a second step, a first pass effect was included in the model to account for the higher norbuprenorphine exposure after OTM administration when compared with other routes (Fig 2). The rate constant of transformation from parent to metabolite was estimated using the IV and SC datasets (Ka_Met_ = 0.196 hour^-1^, 19%) yielding a transformation half-life 3.5 hours. The Ka_Met_ was then fixed to allow estimation of the first pass norbuprenorphine absorption rate constant using full 3-routes dataset (Ka_first-pass_ 0.626 hour^-1^, 6.9%, absorption half-life 1.1 hours) and F_OTM_ (parent and metabolite OTM bioavailability). The amount absorbed as norbuprenorphine via first pass after OTM administration contributed to approximately 1% out of the value of F_OTM_ (23.6%).

PK-PD modeling—Several models were evaluated (S3 File—Appendix 3) and the best one was selected based on the Bayesian Information Criterion value and the identifiability of parameters. The model could reliably estimate the effect of buprenorphine but not that of norbuprenorphine. A hypothetical effect-compartment accounted for the delay in attaining maximal effect in relation to drug concentrations in the central compartment [21]. The link between effect site buprenorphine concentration (C_e_) and thermal antinociceptive effect (E) was modelled with an E_max_ function according to equation 1:

$$Antinociceptive\;effect\;\left(E\right)=T_{0}+\;\frac{E_{max}\times C_{e}^{\;n}}{EC_{50}^{\;n}+\;C_{e}^{\;n}}$$

where T_0_ is the estimated baseline thermal threshold (°C), E_max_ is the estimated maximal effect (°C), EC_50_ is the plasma concentration achieving 50% of E_max_ and n is the slope parameter of the concentration effect curve. The transfer rate constant Ke0 was 0.52 hour^-1^ (IIV 0%). The estimated maximal effect E_max_ was 5.62°C (IIV 0%) over the baseline thermal threshold T_0_ = 46.1 (IIV 0%). Buprenorphine EC_50_ (potency) was 2.13 ng mL^-1^ (IIV 447%) with a slope (n) of 1.54 (91%).

## Discussion

Subcutaneous administration of the drug provided long-lasting thermal antinociception (≥ 24 hours). These effects were prolonged compared with the IV (8 hours) and OTM (≥ 8 hours) treatments. The combined modelling approach provided a robust model to capture the complex absorption of the SC treatment. Despite limitations in the thermal antinociceptive model such as right censored data (due to the safety cut-off) and individual variability, the final PK-PD model could describe and predict the prolonged analgesic effects of Simbadol^™^ after SC administration due to is biphasic rapid and slow absorption kinetics.

The duration of thermal antinociception was consistently longer in this study compared with traditional doses of buprenorphine, regardless of route of administration. In previous studies that tested thermal antinociception of buprenorphine in cats, antinociception was reported between 4 and 12 hours following 0.01 mg kg^-1^ IM buprenorphine [8]. Further investigation, including IV and OTM administration at doses of 0.01–0.02 mg kg^-1^ found shorter durations of antinociception (between 0.5 to 6 hours) [9, 12–14, 18, 22]. Additionally, it appeared that low dose buprenorphine administered SC was ineffective, with near undetectable plasma concentrations that did not provide thermal antinociception [18]. Clinical guidelines would not recommend the SC route of administration for the management of pain using low doses of buprenorphine [1]. In the current study, the increased dose of buprenorphine prolonged the duration of antinociceptive effect in all three routes of administration, however it is clear that the SC route of administration provided superior duration when compared with all others. A recent study compared thermal antinociceptive effects of buprenorphine using different doses (0.02–0.24 mg kg^-1^) and found thermal antinociception of up to 30 hours when 0.12 mg kg^-1^ or more was administered [3]. In the study herein, the SC administration produced consistent elevations in TT when compared with either baseline or placebo treatments.

Joint modeling provided robust estimates of the pharmacokinetic parameters of Simbadol^™^ for several reasons. Traditional methods used for pharmacokinetic modeling of buprenorphine in cats have studied each route of administration separately [9, 16, 18], instead of pooling data from crossover studies to strengthen estimation of parameters shared across routes of administration (CL, V1, CL2, V2). In the present study, six individuals were used in a typical cross-over design, yielding 18 related (six cats, three routes of administration) plasma concentration-time curves. Amalgamation of rich PK data from different routes in the same model using non-linear mixed effect modelling is encouraged to optimize data, and increase statistical power to address complex absorption or disposition kinetics [4]. In the present study, the slow SC absorption kinetic could be unraveled due to the inclusion of IV data in the model (during exploratory deconvolution exercise or in the final model), which is a crucial requirement to study atypical drug absorption profiles [23]. The slow SC absorption resulted in a delayed T_max_ compared with the OTM route. However, the beta elimination half-life of 12.3 hours was still longer than the half-life of the slowest SC absorption input (11.2 hours for TD input), therefore ruling out a flip-flop phenomenon.

The kinetic of norbuprenorphine followed closely the kinetic of the parent drug. Not only peak norbuprenorphine concentrations appeared much earlier after IV or OTM when compared with SC administration, but the plasma norbuprenorphine concentration-time curve also displayed double peaks corresponding to the one observed for buprenorphine. Hence, joint modelling of buprenorphine-norbuprenorphine further corroborated the slow biphasic SC buprenorphine uptake using a robust model.

The biphasic input model used has been reported in a previous study where a double peak phenomenon in the plasma concentration-time curve of ropivacaine after femoral blockade was observed in people [24]. After a rapid short-lasting absorption from the perineural space, the secondary slower input was accounted by either (i) partitioning of the drug in the surrounding tissues and subsequent mobilization with exercise-induced increase in perfusion or (ii) initial precipitation in surrounding tissues due to a low aqueous solubility and gradual re-dissolution, creating a concentration gradient that promotes systemic absorption (16). It is possible that a similar process occurs in the cat following SC administration of Simbadol^™^.

The pharmacokinetic parameters estimated in the current study are similar to those reported in the literature (Table 3) using buprenorphine in cats [9, 16, 18, 25]. Some variability exists due to different bioavailability particularly in routes other than IV. For example, the elimination t_1/2_ was longer in the current study, yet the clearance and VD_SS_ are similar across studies. One potential limitation in the present study is the variable doses between OTM/IV and SC (0.12 mg kg^-1^ vs 0.24 mg kg^-1^). In a study from Taylor et al, increasing dosages of buprenorphine (0.02 to 0.24 mg kg^-1^ SC) revealed saturation kinetics (Table 4) (3). Similar clearances were observed, however the lack of IV administration in the latter study does not allow for an estimate of bioavailability, making VD_SS_ comparisons difficult. Indeed, the elimination half-life was shorter in the current study when compared with Taylor et al. This is likely a result of the variable formulations used in that study, which might alter drug absorption.

**Table 3:** Median pharmacokinetic estimates according to different dosage regimens and studies.

Parameter	Units	Current Study	Hedges et al. 2014		Steagall et al. 2013			Robertson et al. 2003	Taylor et al. 2001	
Dose	μg kg^-1^	120–240	20					10		
Route		All routes	IV	OTM	IV	IM	SC	OTM	IV	IM
Clearance or CL/F	L kg^-1^ hour^-1^	0.98	1.4	3.8*	0.5	0.8*	-	0.51*	1	1.4*
V_D-Steady State_	L kg^-1^	7.9	11.6	25.9	2.9	10.3	-	3.4	7.1	8.9
Elimination half-life	Hour	12.3	9.8	8.9	7	7.7	-	5.8	6.9	6.3

*Values converted from published estimates to standardize units*. Intravenous (IV), Buccal (B), Intramuscular (IM), Subcutaneous (SC).

*Variable bioavailability F%.

**Table 4:** Median pharmacokinetic estimates using high doses of buprenorphine by different routes of administration in cats.

Parameter	Units	Current Study	Taylor et al. 2015			
Dose	μg kg^-1^	120–240	20	60	120	240
Route		All routes	SC	SC	SC	SC
Clearance or CL/F	L kg^-1^ hour^-1^	0.98	-	1.0	0.92	0.94
V_D-Steady State_	L kg^-1^	7.9	-	0.58*	0.59*	0.66*
Elimination Half-life	Hour	12.3	2.7	22.4	19.7	17.2

*Values converted from published estimates to standardize units, Subcutaneous (SC)*,

*Vdbeta/F (L/mL)

Current literature on OTM or sublingual administration of buprenorphine has suggested that jugular sampling may be inappropriate for PK studies using this drug because it overestimates bioavailability due to sampling a vessel which drains the site of administration [26]. There are two studies in particular in which this overestimation has been documented. One study found an F of 116% and the other 139% in cats [9] and horses [27], respectively. When carotid arterial, jugular venous and saphenous sampling sites were simultaneously compared in cats, F was reported as 32%, 47% and 23% respectively [26]. These factors were considered in the design of the current study. However, placing a catheter in the carotid artery three times during the study and for prolonged periods was found to be risky with a potential for blood sampling failure. The authors chose to place a central venous catheter to collect samples from a central site, rather than peripheral venous site. A pilot study showed that placing these catheters via the jugular site (versus medial saphenous) was both easy and repeatable, and the sampling port would require minimal restraint of the cats. As a further step to minimize overestimation, cats receiving OTM treatment would have the dose administered in the contralateral cheek relative to the jugular catheter. The reported bioavailability of the OTM treatment in the current study (F = 23.6%) is closer to that of the peripheral venous sample obtained in the previous study [26].

The SC route of administration provided prolonged analgesia due to its sustained plasma concentrations and the relationship between plasma, effect-side concentrations and time course of antinociception predicted by the sequential population PK/PD model. Despite consistent thermal antinociception based on averaged group data, the fit of the pharmacodynamic model was less satisfactory on an individual basis. Great individual variability in response to thermal stimulation was observed, however, similar findings have been reported (16). Treatments produced significant and variable behavioral and TT changes that could have affected the quality of the PK/PD model fit.

Other limitations in the present study were the inclusion of a safety threshold that produces artificially truncated data. A log likelihood approach to model right censored data as proposed by Sadiq et al. [28] was attempted but did not allow successful modelling with Phoenix NLME. In addition, PD modelling has not been described for norbuprenorphine in cats. In other species, the norbuprenorphine is between 50 and 200-fold less potent than buprenorphine with regards to its respiratory depressant or antinociceptive effects [29]. Multiple models including both drug and metabolite were attempted to evaluate the contribution of norbuprenorphine to thermal antinociception (S3 File—Appendix 3), however the PD parameters for norbuprenorphine were not identified.

## Conclusion

Subcutaneous administration of Simbadol^™^ (Buprenorphine HCl, 1.8 mg ml^-1^) provided long-lasting thermal antinociception (≥ 24 hours) in conscious cats. These effects are prolonged compared with the IV (8 hours) and OTM (≥ 8 hours) treatments. Joint pharmacokinetic-pharmacodynamic modelling showed prolonged plasma concentrations for the SC route. Despite the difficulties with pharmacodynamic modelling, the final model strongly supported the long acting analgesia provided by the drug. Advanced mathematical modelling of pooled data from different routes or parent-metabolite drug combinations and different studies allows leveraging of information to improve the understanding of complex pharmacokinetics.

## Supporting information

S1 File Acclimatization of cats. (DOCX)

S2 File Analytical methods for buprenorphine and norbuprenorphine. (DOCX)

S3 File Population pharmacokinetic-pharmacodynamic modelling. (DOCX)

S4 File Raw data. (XLSX)
