# Supplementary material for: Pharmacokinetic and pharmacodynamic modelling after subcutaneous, intravenous and buccal administration of a high-concentration formulation of buprenorphine in conscious cats
Source: PLoS One. 2017 Apr 26;12(4):e0176443. doi: 10.1371/journal.pone.0176443 (PMC5405979; doi:10.1371/journal.pone.0176443)
Supplement: S1 File — (DOCX) [file pone.0176443.s001.docx]

**Appendix 1 – Acclimatization of cats**

Cats were moved daily (Monday to Friday) into the laboratory room and housed individually in the cages for the study. The threshold device (see section on Thermal threshold testing) was applied to the cats, but not tested, for one and a half hours. The cats were then allowed to rest for another half hour in the cage. During the second week of acclimatization (Monday-Friday), the response to thermal stimulation for each cat was studied every 30 minutes during a one-hour period (two readings) with the stimulus applied and tested. This was done to familiarize the observer (BM) with the cat’s individual responses. During testing and acclimatization, cats were free-ranging and unrestrained at all times. Water, toys and a litter box were available at all times during testing and acclimatization. Food was offered between testing periods. Two weeks before acclimatization, cats were visited by one observer (BM/GD/PS) on a daily basis (Monday-Friday) for at least 15 minutes. By the time that the study began, the cats had lived in the accommodation for at least one month.
