# Supplementary material for: Pharmacokinetic and pharmacodynamic modelling after subcutaneous, intravenous and buccal administration of a high-concentration formulation of buprenorphine in conscious cats
Source: PLoS One. 2017 Apr 26;12(4):e0176443. doi: 10.1371/journal.pone.0176443 (PMC5405979; doi:10.1371/journal.pone.0176443)
Supplement: S2 File — (DOCX) [file pone.0176443.s002.docx]

**Appendix 2 – Analytical methods for buprenorphine and norbuprenorphine**

Plasma samples were analyzed for buprenorphine and norbuprenorphine using HPLC-MS/MS (High Performance Liquid-chromatography tandem mass spectrometry). The drug and its metabolite were extracted using a liquid-liquid preparation technique. A total of 250 µL of standard solution (10.0 ng mL^-1^ ^2^H_3_-norbuprenorphine and ^2^H_4_-buprenorphine) was added to 100 µL of sample. This mixture was alkalinized using 50 µL of 5M ammonium hydroxide in a borosilicate sample tube. The sample was then vortexed for five seconds. Four millilitres of ethyl acetate were added and the sample mixed again via rotation for 20 minutes. All samples were then centrifuged at ~3500 *g* for 10 minutes and the organic layer transferred into a new sample tube. The samples were evaporated at 50°C under a stream of nitrogen. The dry residues were resuspended with 100 µL of 20:80 (v/v) methanol:water solution and transferred into injection vials for analysis. An isocratic mobile phase was used with a Phenomenex PFP(2) (150 x 3 mm I.D., 3 µm) and PFP (4 x 2.0 mm) security guard cartridge operating at 50°C. The mobile phase consisted of acetonitrile and 1.0% (v/v) formic acid in type 1 water at a ratio of 50:50, respectively. The flow rate was fixed at 0.30 mL min^-1^ and norbuprenorphine, buprenorphine and their respective internal standards (^2^H_3_-norbuprenorphine and ^2^H_4_-buprenorphine) eluted at three and four minutes, respectively. Five µL of the extracted sample was injected and the total run time was set to five minutes. The mass spectrometer (MS) was interfaced with the high-performance liquid chromatography (HPLC) system using a pneumatic assisted heated electrospray ion source. MS detection was performed in positive ion mode, using selected reaction monitoring (SRM). Nitrogen was used for the sheath and auxiliary gases and was set at 50 and 15 arbitrary units. The HESI electrode was set to 3500 V. The capillary temperature was set at 350°C. Argon was used as collision gas at a pressure of 2.5 mTorr. Total cycle time was set at 0.25 seconds. Peak width of Q1 and Q3 were both set at 0.7 FWHM. Analysis of the sample assay method met standards for generally accepted bioanalytical chemistry, as such, sample analysis was performed (1). The result was the development of a HPLC-MS/MS method which was validated for determination of feline plasma analysis for buprenorphine and norbuprenorphine.

1. CDER, CVM. Guidance for Industry: Bioanalytical method validation Rockville, MD: Food and Drug Administration; 2013. Available from: <http://www.fda.gov/downloads/drugs/guidancecomplianceregulatoryinformation/guidances/ucm368107.pdf>.
