# Supplementary material for: Pharmacokinetic and pharmacodynamic modelling after subcutaneous, intravenous and buccal administration of a high-concentration formulation of buprenorphine in conscious cats
Source: PLoS One. 2017 Apr 26;12(4):e0176443. doi: 10.1371/journal.pone.0176443 (PMC5405979; doi:10.1371/journal.pone.0176443)
Supplement: S3 File — (DOCX) [file pone.0176443.s003.docx]

**Appendix 3: Population pharmacokinetic-pharmacodynamic modelling**

A classic two compartment model with first order absorption was the starting point for modelling of the 3 routes simultaneously. It showed acceptable fitting for the IV and OTM routes, but failed to capture the multiple peaks displayed on the SC plasma concentration-time curve of all cats.

**Preliminary deconvolution analysis:**

To explore the complex absorption of buprenorphine SC, a deconvolution procedure was initially carried out to evaluate the shape of the SC input function over time (1). The PK disposition parameters from the IV fitting of the present study were used for this purpose. The biphasic input consisted of an initial sharp burst of absorption followed by a slower release phase. Therefore, a series of dual input models were tested to characterise the consistent double peak phenomenon observed after SC administration.

**Goodness of fit:**

For each Phoenix NMLE run, goodness of fit plots were prepared (2). The nested candidate models were compared on the basis of their biological plausibility, prediction based diagnostics (PRED, IPRED), residual-type diagnostics (RES and IRES), simulation based diagnostics (stratified VPC) and numerical diagnostics (minimisation of the Objective Function Value (OVF)). These were statistically tested with the Likelihood Test Ratio (when LRT was performed, deltaOVF >6.64; P<0.01, df = 1, otherwise the Akaike Information Criterion (AIC) was used) as along with measures of model stability and adequacy (convergence, precision of the parameters estimates).

**Statistical description of the model:**

Inter-animal variability was characterised with the assumption individual parameters were log-normally distributed around the population value (Eq. 1):

$P_{ij}= \theta_{j} \times exp(\eta_{ij})$ (1)

Where P_ij_ is the *j*-th parameter value for individual *i*; θ_j_ is the typical value for the *j*-th parameter for the population; and η_ij_ is normally distributed around 0 with a variance of ω^2^_j_. To minimise the residual variability (difference between predicted and observed values), additive and proportional error models were compared.

Parameters bound between 0 and 1 (typically bioavailabilities, noted F) were expressed and estimated in the model after a logit transform. The typical value of F (θ_F) was then used in equation 2 to yield a final estimate.

$F_{i}=inv logit (\theta_{F}+ {\eta F}_{i})$ (2)

Where F_i_ is the inverse logit of θF, the typical value of the bioavailability, and ηF_i_ is the residual for the i^th^ invidual.

The coefficient of variation of the PK parameters were approximated as follows (Eq. 3):

$CV\left( \% \right)= \sqrt{exp \left( \omega^{2} \right)-1} \times100\%$ (3)

Visual predictive checks were built to evaluate the performance of the final model by comparing the median of simulated (n=5000) plasma concentrations with observed data (+/- 5^th^ and 95^th^ percentiles).

**PK modelling**

**Base model development for the SC administration**

First, a 2 compartment model was composed to simultaneously fit the IV and the OTM. This allowed estimation of the physiological PK parameters common to the three routes of administration (namely CL, total body clearance; V, volume of the central compartment; CL2, intercompartmental clearance and V2, volume of the peripheral compartment). The model also provided the OTM absorption rate constant (ka_OTM_) and absolute OTM bioavailability (F_OTM_). The typical value θ_j_ and individual η_ij_ were fixed, thus reducing the number of parameters estimated in the complex modelling of SC absorption.

Zhou (3) reviewed strategies to model atypical absorption profiles. Several of these models were tested in combination to capture the double peak phenomenon (table 1). The best model was the from Gaudreault et al. (1) which combined an Inverse Gaussian (IG) input function and a time dependent (TD) input function.

**Table1**: Comparison of rival models for SC input function in joint IV, OTM and SC buprenorphine model and selection of best model

| **Combination for SC input function** | **OFV (-2LL)** | **AIC** | **Comment** |
| --- | --- | --- | --- |
| Single FO Absorption | 646 | 680 | No capture of the second peak |
| Combination of two FO absorptions | 626 | 644 | Double peak not captured |
| Combination of two FO absorptions with tlag | 599 | 621 | Can capture double peak but misfit |
| Combination of two ZO absorptions with tlag | 630 | 652 | Double peak not captured |
| IG combined with FO absorptions with tlag | 512 | 538 | Good peak captures but FO restricts flexibility in late phase |
| Combination of two IG | 644 | 670 | Good initial peak capture but constant bias in late phase (prediction under-estimates concentrations) |
| IG combined with TD (gamma fixed) | 498 | 526 | Best fitting but identifiability issues and some peak misfits |
| ***IG combined with TD (gamma = CL)*** | ***505*** | ***531*** | ***Best model: Excellent peak capture, predictions slightly under-estimate concentrations*** |

FO: first order, ZO: zero order, IG: inverse Gaussian input function, TD: time dependent input function.

First, a combination of two first order absorption phases were evaluated together to account for the final SC bioavailability (F_SC_). The parameter BIO (value bound between 0 and 1) defined the proportion of the dose mobilised by the first process and consequently the proportion of the dose mobilised by the second process (1-BIO).

First order input was modelled as in equation 4:

$I\left( t \right)=Dose \times F \times exp (-k_{a}\times t)$ (4)

Second, an Inverse Gaussian distribution was evaluated to model the early and sharp peak of the buprenorphine input function (4). The density function of the Inverse Gaussian input can be written as a time-dependent function according to the following equation (Eq. 5):

$I\left( t \right)=F_{1 \cdot} \left[ \frac{MAT}{2\pi{\times CV}^{2} \times t^{3}} \right]^{\frac{1}{2}}\cdot exp \left[ -\frac{{(t - MAT)}^{2}}{2 {\times CV}^{2} \times MAT \times t} \right]$ (5)

With the 3 following parameters estimated: mean input rate time (MAT, representing the mean of the distribution), the variance of the input time distribution (CV^2^, representing the skewness of the distribution) and the proportion of the SC bioavailability absorbed by this early process.

The mode of the IG distribution (t_max_) is the time at which the input rate reaches its maximum. This was calculated as a more informative secondary parameter in order to facilitate interpretation of the values of MAT and CV^2^ (4) (Eq 6):

$t_{max}=MAT \times\left[ \sqrt{1+ \frac{9}{4} \times{CV}^{4}} - \frac{3}{2} \times{CV}^{2} \right]$ (6)

Third, Inverse Gaussian with time dependent absorption as in equation 7:

$I\left( t \right)=\frac{F . Ka\text{ }}{[1+e^{-\left( \frac{t -t50}{\gamma} \right)}]}$ (7)

Where k_a_ is the classic first-order absorption rate constant; t_50_ is the time required to achieve 50% of the maximal input rate; and γ is a shape parameter for the time-dependent process. Gamma (γ) was approximated by the value of clearance, as performed by Gaudreault et al.(1),

**Pharmacodynamic modelling**:

The full PK model (metabolite and parent drug) served as base for the sequential PKPD modelling. The different pharmacodynamic rival models, with or without the effect of the metabolite (norbuprenorphine), were compared (Table 2).

Of the four best models, none fit the data exceptionally well. The most complicated included 2 to 4 additional parameters which provided a slightly better fit, but were less identifiable than the most parsimonious model. The analgesic effect of norbuprenorphine in cats is unknown, this absence likely impacted the final PD model.

Table 2: final comparison of the 4 best PD models.

| **Pharmacodynamic model comparison (all with log normally distributed parameters and additive error model)** | **OFV (-2LL)** | **AIC** | **BIC** | **#Para-meters** | **Comment** |
| --- | --- | --- | --- | --- | --- |
| ***A) Buprenorphine only: Effect compartment (Ke0) and Sigmoid Emax*** | ***1881.1*** | ***1907.1*** | ***1966.8*** | ***13*** | ***Best model*** |
| B) Buprenorphine only: Same model as above but inclusion of 3 equilibrations constants (Ke0): 1 for each route | 1872.1 | 1906.1 | 1984.3 | 17 | 3 Ke0 physiologically unrealistic |
| C) Buprenorphine and Norbuprenorphine: Effect compartment model with competitive agonism, sharing the same Emax and Ke0 but with constraints on relative potency (different EC50) | 1877.5 | 1907.5 | 1976.4 | 15 | Better fitting but unrealistic estimates |
| D) Buprenorphine and Norbuprenorphine: same model as above but allowing 2 different constants for effect compartment Ke0 and Ke0m (metabolite) | 1863.3 | 1897.3 | 1975.5 | 17 | Unstable model |

-2(LL): objective function value, AIC: Akaike Information Criterion, BIC: Bayesian Information Criterion

Ultimately the most parsimonious model (A) was adopted based on the BIC and the conservative identifiability. The absence of PK/PD data after administration of norbuprenorphine only precludes estimation of norbuprenorphine PD parameters in a joint model when taking into account the effect combined antinociceptive effect of parent drug and metabolite.

Model A is an effect compartment model with sigmoid E_max_ expression. In this model, a hypothetical pharmacodynamic compartment accounts for the delay in attaining maximal effect in relation to drug concentrations in the central compartment (5). The assumptions behind the effect-compartment model were: (i) first order processes (k_e0_ transfer rate constant for buprenorphine,) govern the onset and offset of the pharmacodynamics effect; (ii) the amount of drug in the effect compartment is negligible and does not affect plasma concentration, and (iii) the concentration in the effect compartment (C_e_) and plasma are equal at steady state.

The link between effect site buprenorphine concentration (C_e_) and plasma buprenorphine concentration was modelled with a differential equation (8):

$\frac{{dC}_{e}}{dt}= {ke}_{0} \times(C- C_{e})$ (8)

Where Ke0 is the equilibration rate constant between the central and the effect compartment.

The thermal antinociceptive effect (E) was modelled with an E_max_ function according to equation 9:

$Antinociceptive effect (E)=T_{0}+ \frac{E_{max}\times C_{e}^{n}}{{EC}_{50}^{n}+ C_{e}^{n}}$ (9)

Where T_0_ is the estimated baseline thermal threshold (°C); E_max_ is the estimated maximal effect (°C); EC_50_ is the plasma concentration achieving 50% of E_max_; and n is the slope parameter of the concentration-effect curve.

**LL models for right censored data**

Due to safety cut-off (55 °C ), the true TT for a number of timepoints would have been above this artificial limit to the nociceptive test (right censored data). Attempts to fit models which take into account right censored data were not successful (using log likelihood approach) This is a common problem in antinociception studies (6) and the implementation of this modelling approach is much easier in Monolix with the SAEM algorithm.

**Results and goodness of fit plots:**

The goodness of fit figures for the final PK model fitting (buprenorphine and metabolite) are included thereafter:

- Fig suppl. 1: observed values vs population prediction,
- Fig suppl. 2: observed values vs individual predictions,
- Fig suppl. 3: conditional weighted residuals vs time after dose,
- Fig suppl. 4: conditional weighted residuals vs population prediction,
- Fig suppl. 5 a. to f.: visual predictive check for buprenorphine IV (5.a), norbuprenorphine IV (5.b), buprenorphine OTM (5.c), norbuprenorphine OTM (5.d), buprenorphine SC (5.e), norbuprenorphine SC (5.f)

The goodness of fit figures for the final PD model fitting (buprenorphine only) are included thereafter:

- Fig suppl. 6: observed values vs population prediction,
- Fig suppl. 7: observed values vs individual predictions,
- Fig suppl. 8: conditional weighted residuals vs time after dose,
- Fig suppl. 9: conditional weighted residuals vs population prediction,
- Fig suppl. 10 a. to c.: pharmacodynamic visual predictive check for buprenorphine IV (10.a), OTM (5.b), buprenorphine SC (5.c)

The average trend (50^th^ percentile) of the data was well captured for the IV and SC initially. None of the models really captured the fast onset and intensity of OTM and all models tended to estimate the duration of analgesia beyond its real duration with SC.

Fig suppl. 1 (observed values vs population predictions)

Fig suppl. 2 (observed values vs individual predictions)

Legend: CObs_A_IV: buprenorphine after IV administration, CObsMet_A_IV: norbuprenorphine after IV administration, CcObs_B_OTM: buprenorphine after OTM administration, CcObsMet_B_OTM: norbuprenorphine after OTM administration, CcObs_C_SC: buprenorphine after SC administration, CcObsMet_C_SC: norbuprenorphine after SC administration, DV = dependent variable (observed value), PRED = population predictions, IPRED = individual predictions

Fig suppl. 3 (conditional weighted residuals vs time after dose)

Fig suppl. 4 (conditional weighted residuals vs population prediction)

Legend: CObs_A_IV: buprenorphine after IV administration, CObsMet_A_IV: norbuprenorphine after IV administration, CcObs_B_OTM: buprenorphine after OTM administration, CcObsMet_B_OTM: norbuprenorphine after OTM administration, CcObs_C_SC: buprenorphine after SC administration, CcObsMet_C_SC: norbuprenorphine after SC administration, CWRES = conditional weighted residual, PRED = population predictions

Fig suppl. 5a: Stratified Visual Predictive Check (buprenorphine IV PK)

Fig suppl. 5b: Stratified Visual Predictive Check (norbuprenorphine IV PK)

Fig suppl. 5c: Stratified Visual Predictive Check (buprenorphine OTM PK)

Fig suppl. 5d: Stratified Visual Predictive Check (norbuprenorphine OTM PK)

Fig suppl. 5e: Stratified Visual Predictive Check (buprenorphine SC PK)

Fig suppl. 5f: Stratified Visual Predictive Check (norbuprenorphine SC PK)

- Fig suppl. 6: observed PD values vs population prediction,
- Fig suppl. 7: observed PD values vs individual predictions,

- Fig suppl. 8: conditional weighted residuals on PD vs time after dose,

- Fig suppl. 9: conditional weighted residuals on PD vs population prediction,

- Fig suppl 10.a: Stratified VPC: Pharmacodynamics fitting IV (DV0 = dependent variable = thermal threshold)

- Fig suppl 10.b: Stratified VPC: Pharmacodynamics fitting OTM
- Fig suppl 10.c: Stratified VPC: Pharmacodynamics fitting SC

References

1. Gaudreault F, Drolet P, Fallaha M, Varin F. A population pharmacokinetic model for the complex systemic absorption of ropivacaine after femoral nerve block in patients undergoing knee surgery. J Pharmacokinet Pharmacodyn. 2012;39(6):635-42.

2. Karlsson MO, Savic RM. Diagnosing model diagnostics. Clin Pharmacol Ther. 2007;82(1):17-20.

3. Zhou H. Pharmacokinetic strategies in deciphering atypical drug absorption profiles. J Clin Pharmacol. 2003;43(3):211-27.

4. Csajka C, Drover D, Verotta D. The use of a sum of inverse Gaussian functions to describe the absorption profile of drugs exhibiting complex absorption. Pharm Res. 2005;22(8):1227-35.

5. Sheiner LB, Stanski DR, Vozeh S, Miller RD, Ham J. Simultaneous modeling of pharmacokinetics and pharmacodynamics: application to d-tubocurarine. Clin Pharmacol Ther. 1979;25(3):358-71.

6. Sadiq MW, Bostrom E, Keizer R, Bjorkman S, Hammarlund-Udenaes M. Oxymorphone active uptake at the blood-brain barrier and population modeling of its pharmacokinetic-pharmacodynamic relationship. Journal of pharmaceutical sciences. 2013;102(9):3320-31.
